# Durability of Parameters Associated With Endurance Running in Marathoners

**DOI:** 10.1002/ejsc.70073

**Published:** 2025-10-23

**Authors:** Ben Hunter, Daniel Muniz‐Pumares

**Affiliations:** ^1^ School of Human Sciences London Metropolitan University London UK; ^2^ School of Health, Medicine, and Life Sciences University of Hertfordshire Hatfield UK

**Keywords:** endurance performance, fatigability, physiological testing, resilience

## Abstract

Physiological markers of endurance performance include the maximal oxygen uptake (V̇O_2peak_), its fractional utilisation at lactate threshold (FU_LT_), and running economy (RE), which are closely tied to the speed eliciting the lactate threshold (sLT). These parameters deteriorate during prolonged exercise, and the ability to resist such declines (i.e., durability) is now also considered a marker of marathon performance. This study investigated the durability of markers of endurance performance (V̇O_2peak_, FU_LT_ and RE), and whether the durability of these markers was associated with marathon performance. Eighteen participants of the 2024 London Marathon (11 males, age: 41 ± 12 years, marathon finish time: 3:17 ± 0:32 h:min) completed two separate visits to determine V̇O_2peak_, FU_LT_, RE and sLT in a ‘fresh’ state (PRE) and following a 90‐min run at sLT (POST). Reductions in V̇O_2peak_ (PRE: 56.7 ± 7.2 mL·kg^−1^·min^−1^ vs. POST: 53.4 ± 6.3 mL·kg^−1^·min^−1^, *p* < 0.001) and sLT (PRE: 12.8 ± 2.0 km·h^−1^ vs. POST: 12.1 ± 2.2 km·h^−1^, *p* < 0.001) were evident, but RE and FU_LT_ were unchanged (both *p* > 0.05). The percentage change in sLT between POST and PRE (*r* = 0.680, *p* < 0.01) was significantly associated with marathon performance, whereby small deteriorations of sLT were associated with faster marathon times. Prolonged running impairs key physiological markers of endurance performance, and the degree of this deterioration, that is, durability, is associated with marathon performance. Marathon runners and practitioners should consider quantifying durability to complement existing physiological markers.

## Highlights

- Peak oxygen uptake and parameters associated with the lactate threshold are subject to deterioration following prolonged running.
- The durability of parameters associated with the lactate threshold, including the relative V̇O_2_ at lactate threshold and the speed at lactate threshold, are associated with marathon performance.
- Durability should be considered alongside traditional performance markers to improve physiological profiling in marathon runners.

## 1 Introduction

Thousands of recreational runners take part in mass events every year and professional athletes continue to pursue and break records, facilitated in part by technological developments and better running practices (Haugen et al. 2022; Hoogkamer et al. 2017; Joyner et al. 2020; Santos‐Concejero et al. 2020). It is widely recognised that physiological characteristics can explain much of the variation in endurance performance. Specifically, physiological models of endurance performance typically consider the maximal oxygen consumption (V̇O_2max_), the fraction utilisation of V̇O_2max_, typically constrained by physiological landmarks such as the lactate threshold (LT) or lactate turnpoint, which combined results in a ‘performance’ V̇O_2_, and the ability to convert such V̇O_2_ into a mechanical output, quantified as running economy (RE) (Jones et al. 2021; Joyner and Coyle 2008). These three physiological traits are considered the ‘big‐three’, as combined can predict 72.1% of the variability in marathon performance (di Prampero et al. 1986). However, there is substantial evidence that these markers deteriorate during prolonged exercise (Brueckner et al. 1991; Clark et al. 2018; Hamilton et al. 2024; Hunter et al. 2025; Nuuttila et al. 2024; Stevenson et al. 2022; Unhjem 2024; Zanini et al. 2024). A key observation is that durability, which reflects the extent to which these markers of endurance performance deteriorate during prolonged exercise (Maunder et al. 2021), is heterogeneous. It has been suggested that the magnitude of this deterioration may influence endurance performance (Hunter et al. 2025; Jones 2023).

The deterioration in RE following, or during, prolonged running is well documented (Brueckner et al. 1991; Xu and Montgomery 1995; Zanini et al. 2024). However, relatively little attention has been paid to the change in physiological thresholds or V̇O_2max_ following prolonged running (Dressendorfer 1991; Hunter et al. 2025; Unhjem 2024; Zanini et al. 2025). V̇O_2max_ has been shown to decrease by 6%–7% following 90 min of running (Dressendorfer 1991; Zanini et al. 2025), but not following a 60‐min run at 70% V̇O_2max_ (Unhjem 2024). When considering physiological thresholds, Nuuttila et al. (2024) and Zanini et al. (2025) have demonstrated a deterioration of the speed at LT (sLT) following running. Similar findings have been demonstrated to the power output and V̇O_2_ associated with VT_1_ and LT in cycling (Gallo et al. 2024; Hamilton et al. 2024; Stevenson et al. 2022, 2024). The downward shift in physiological thresholds is of relevance to marathon running, as a premature transition into a higher intensity domain would result in markedly different physiological responses (e.g. Black et al. 2017), thus expediting fatigue and reducing performance. Limited understanding is currently available on changes to the fraction of V̇O_2max_ associated with LT (FU_LT_) following prolonged exercise. However, a recent study showed an increase in FU_LT_ (Zanini et al. 2025), although this was largely due to depressed V̇O_2max_ values, and thus not a true increase in sustainable exercise capacity. Given the close association between each of these parameters and marathon performance (Jones et al. 2021; Joyner 1991), a deterioration in any of these markers following prolonged running will negatively impact at least part of the marathon. However, limited research has aimed to examine durability as a determinant of performance in marathon or ultramarathon running events (De Pauw et al. 2024; Smyth et al. 2022). Whilst direct physiological measures have not been used, the ratio between internal work rate (e.g., heart rate [HR]) and external work rate (e.g., speed) has been suggested to be an effective field‐based measure of durability, with the potential to differentiate between performance levels (De Pauw et al. 2024; Maunder et al. 2021; Smyth et al. 2022). However, decoupling is subject to cardiac drift, and thus can be affected by environmental conditions and hypovolaemia through sweating and may not entirely represent changes to V̇O_2_, and thus changes to efficiency (Billat et al. 2022; Coyle 1998).

The primary aim of this study was to examine whether the durability of physiological parameters (i.e., V̇O_2max_, FU_LT_, and RE) was associated with marathon performance. Additional aims were to examine the changes to physiological parameters following and during a prolonged exercise, and to assess the time course of HR‐to‐speed decoupling during a marathon. It was hypothesised that: (i) the durability of physiological parameters and marathon performance would be correlated, (ii) physiological parameters associated with marathon performance would decline following prolonged exercise, (iii) physiological parameters during the prolonged run would exhibit significant changes from baseline and (iv) the onset and magnitude of decoupling would be associated with marathon performance.

## 2 Materials and Methods

### 2.1 Participants

Twenty‐three participants gave voluntary written informed consent to take part in the study, after the experimental procedures, risks and potential benefits had been explained. Participants were eligible to take part in the study if they were expected to complete the 2024 London Marathon with expected finish times under 4:00 (h:min) for men and 4:25 for women. All participants who completed the marathon within these times, except for one male who finished in 4:13. The inclusion of this participant in the final analysis did not impact any of the findings, hence was included. All participants were free of recent (< 3 months) musculoskeletal injury and chronic disease and were running > 40 km·week^−1^. The study was performed following the standards of the Declaration of Helsinki except for pre‐registration. Institutional ethical approval was granted before participant recruitment.

### 2.2 Study Design

**FIGURE 1:**
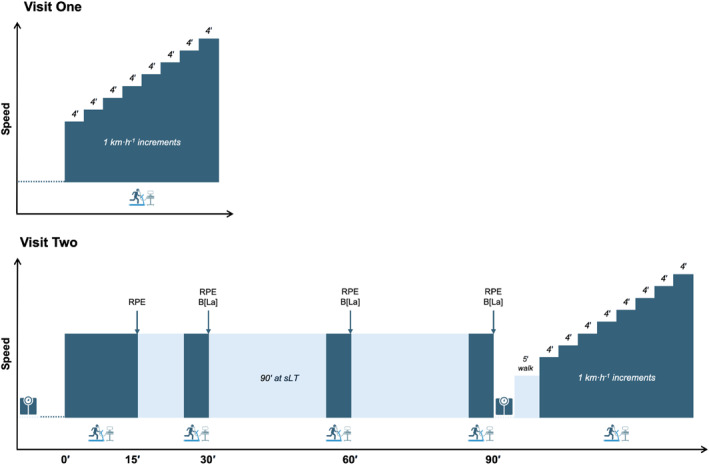
Schematic overview of the testing visits. Areas shaded in dark blue denote the period of pulmonary gas data collection. Arrows denote the collection of rating of perceived exertion (RPE) and capillary blood samples to examine blood lactate concentration (B[La]). For clarity, the time points at which B[La] were sampled during the incremental exercise tests are omitted. Weighing scale icon indicates when measures of body mass were taken in the second visit. Dotted lines denote when participant was at rest.

Participants visited the laboratory on two occasions, separated by a minimum of one day (24 h). The rest between visits was 3.8 ± 3.7 days (range: 1–14 days). The laboratory conditions were monitored during each visit, and no significant differences (both *p* ≥ 0.95) were noted between Visit 1 (temperature: 23.2°C ± 0.9°C, relative humidity: 44.0% ± 4.1%) and Visit 2 (temperature: 23.2°C ± 0.8°C, relative humidity: 43.9% ± 4.9%). The first visit consisted of anthropometric measurements and an incremental exercise test (IET). The second visit consisted of a 90‐min bout of running at sLT, a 5‐min walk at a self‐selected speed, immediately followed by an IET identical to that performed in the first visit (Figure 1). The order of the visits was not randomised, as the parameters derived from the first visit were used to standardise the exercise intensity of the 90‐min run performed during the second visit. Tests were performed within 6 weeks prior to (*n* = 15), or 3 weeks after (*n* = 3, minimum of 10 days after), the London Marathon. Participants were instructed to arrive at both testing sessions fully rested and euhydrated, having consumed 1–2 L of plain water. Testing was conducted at least 2 hours postprandial, and participants were asked to abstain from caffeine for a minimum of 6 hours prior to each session. Participants were advised to keep dietary intake consistent between visits, but dietary intake was not controlled. Each participant started each experimental trial at a similar time of day (± 2 h). All testing was performed on the same motorised treadmill (Woodway 4Front, Woodway, Weil am Rhein, Germany), set at a 1% gradient (Jones and Doust 1996).

### 2.3 Visit One: Characterisation Trial

Upon arrival, participants' stature and body mass were recorded using a stadiometer (Seca 213; Seca Hamburg, Germany) and weighing scale (Seca 803; Seca Hamburg, Germany), respectively. Participants then completed an IET to determine the FU_LT_ and V̇O_2peak_ in a ‘fresh’ state (PRE). The starting speed of the test was chosen to align with approximately 70% of the individual participants' predicted marathon pace (Dantas and Doria 2015), allowing for five to nine stages before task failure. Each stage was 4 min in duration, and the speed was increased by 1 km·h^−1^ until task failure or the participant declined the opportunity to begin the next stage. Task failure was defined as the inability to maintain the criterion speed. A fingertip blood sample was collected within 20 s after each 4‐min stage with the participant straddling the moving treadmill belt. Blood lactate concentration (B[La]) was determined using an automated blood analyser (Biosen C‐Line, EKF Diagnostic, Barleben, Germany). Breath‐by‐breath pulmonary gas exchange data were collected using an online gas analyser (MetaLyzer 3B, Cortex Biophysik, Leipzig, Germany). The participants were fitted with a face mask with known dead space and breathed through a low‐resistance disposable turbine (≤ 0.06 kPa·L^−1^ s^−1^ at 14 L·s^−1^), and the same mask was used for the second visit. A chemical fuel cell and nondispersive infrared sensors were used to measure the concentration of O_2_ and CO_2_. The gas analyser was calibrated before each test with gases of known concentration. The turbine volume transducer did not require calibration prior to use, as the disposable turbine was pre‐calibrated by the manufacturer. HR (H10, Polar Electro Oy, Kempele, Finland) was recorded throughout at 1 Hz.

### 2.4 Visit Two: Prolonged Running Trial

Following the measurement of body mass, participants stood astride the treadmill before lowering themselves onto the moving belt. The time started once the participant let go of the handrails. The participants subsequently completed 90 min at sLT, determined in the first visit (see below for further details). Following the same procedures as visit one, pulmonary gas exchange data were recorded for −3 to 15 min, 25–30 min, 55–60 min and 85–90 min, and HR was recorded throughout. Participants were asked to give their rating of perceived exertion (RPE) using the 6–20 scale (Borg 1998) during the periods of pulmonary gas data collection. Fingertip blood samples were collected within 20 s after the participant straddled the moving belt at rest, 30 min, 60 min and 90 min, and subsequently analysed. Participants were able to consume water ad libitum throughout the prolonged running trial. Following the trial, body mass was assessed and compared to the body mass taken before the trial, and water consumption was quantified to account for sweat loss. Participants then completed 5‐min walking at a self‐selected speed, before commencing an IET, reproducing the procedures described in the first visit to assess parameters in a ‘fatigued’ state (POST). Prior to the IET, a sensor adjustment was performed on the gas analyser to ensure concentration of O_2_ and CO_2_ were measured accurately.

### 2.5 Marathon Performance

During the 2024 London Marathon, participants were asked to record their performance using their own GPS‐enabled watch or phone and HR monitor. Temperature and relative humidity on the day of the Marathon were 10°C and 54.8%, respectively. No advice regarding pacing, nutritional or fluid intake strategies was provided to the participants, and, except for pacing, these parameters were not evaluated. Following the conclusion of the marathon, participants were asked to submit fit files for subsequent analysis. The speed and HR were extracted, and averaged into 5‐km bins, plus the final 2.2 km. The speed extracted from the race files were verified using the 5‐km split times from the 2024 TCS London Marathon website (https://results.tcslondonmarathon.com/2024/). As an indirect estimate of durability during the marathon, the ratio between internal‐to‐external work rate was calculated for each 5‐km bin. Internal work rate was given as HR as a percentage of the HR at LT measured during the PRE assessment. The external work rate was determined as speed as a percentage of sLT. The decoupling observed in the last 5‐km segment of the race (35–40 km) was used to determine the overall magnitude of the decoupling experienced by each athlete, expressed relative to the 5–10 km segment (Smyth et al. 2022). The onset of decoupling was given as the midpoint of the segment where decoupling exceeded 1.025, corresponding to a 2.5% increase in HR‐to‐work rate ratio (Smyth et al. 2022). Marathon performance was quantified as the average running speed throughout the marathon (km·h^−1^).

### 2.6 Data Analysis

Gas measurements were exported into 10‐s bins following the removal of errant breaths, defined as values lying more than 2 standard deviations (SD) away from the moving mean. V̇O_2peak_ was defined as the highest 30‐s moving average (Martin‐Rincon et al. 2019). Otherwise, gas exchange data (V̇O_2_, V̇CO_2_, rate of ventilation [*V̇*_*E*_], breathing frequency [*F*_*R*_], tidal volume [*V*_*T*_]) and HR from the final minute from each 4‐min stage during the incremental exercise test were extracted for further analysis. Gas exchange data (V̇O_2_, V̇CO_2_, *V̇*_*E*_, *F*_*R*_ and *V*_*T*_) from the final minute of each 5‐min recording period and the first 15 min during the prolonged running bout were also obtained, with the average from the final minute used for further analysis. RE was calculated as the oxygen cost expressed relative to speed (ml·kg^−1^ km^−1^), with the values at 15‐min and 90‐min of the prolonged running trial used for PRE and POST values, respectively.

Body mass measurements recorded prior to the IETs performed in PRE and POST assessments were used to adjust V̇O_2_ at LT and V̇O_2peak_ relative to the appropriate body mass. The FU_LT_ was calculated as the relative V̇O_2_ at LT divided by the relative V̇O_2peak_ in PRE and POST. To account for changes in body mass during the prolonged running bout, the body mass pre‐ and post‐trial was used to create a linear regression to estimate body mass at each time point. This was used to adjust RE and the energetic cost of running throughout the prolonged run. Energy expenditure was calculated in line with Equation (1) (Jeukendrup and Wallis 2005):

(1) $$\text{Energy}\;\text{expenditure}\;\left(\text{kcal}\;\min^{-1}\right)=0.550\;{\dot{V}\text{CO}}_{2}+4.471\;{\dot{V}O}_{2}.$$

Fat oxidation rates and carbohydrate oxidation rates were calculated using Equations (2) and (3), respectively (Jeukendrup and Wallis 2005):

(2) $$\text{Fat}\;\text{oxidation}\;\text{rate}\;\left(g\;\min^{-1}\right)=1.695\;{\dot{V}O}_{2}-1.701\;{\dot{V}\text{CO}}_{2}.$$

(3) $$\text{Carbohydrate}\;\text{oxidation}\;\text{rate}\;\left(g\;\min^{-1}\right)=4.210\;{\dot{V}\text{CO}}_{2}-2.962\;{\dot{V}O}_{2}.$$

The LT was estimated using the Log‐log LT method in both PRE and POST assessments. Briefly, blood [lactate] was plotted against treadmill speed using two segments, and the intersection point of the two lines with the lowest residuals sum of squares taken as the LT (Beaver et al. 1985). The V̇O_2_, energy expenditure, carbohydrate oxidation, fat oxidation, *V̇*_*E*_, *F*_*R*_ and *V*_*T*_ associated with LT were calculated by linear regression of the respective parameter versus speed.

### 2.7 Statistical Analysis

Data are presented as mean ± standard deviation (SD). All statistical analysis was performed using Jamovi Software (Version 2.3.28.0). Normality of data was assessed using the Shapiro‐Wilk test. Paired samples *t*‐test were used to test for differences between PRE and fatigued (POST) measures of sLT, FU_LT_, parameters associated with LT (V̇O_2_, energy expenditure, carbohydrate oxidation, fat oxidation, *V̇*_*E*_, *F*_*R*_, *V*_*T*_), and V̇O_2peak_. A repeated measures one‐way analysis of variance (ANOVA) was used to analyse changes in B[La], RPE, HR, EE, RER, carbohydrate oxidation, fat oxidation, *V̇*_*E*_, *F*_*R*_, *V*_*T*_, RE, and the fractional utilisation of V̇O_2peak_ during the prolonged running bout and for HR, speed, and decoupling during the marathon. A Bonferroni adjustment was used to identify the location of significant differences. Cohen's *d*, with values representing small (*d* = 0.2), moderate (*d* = 0.6), or large (*d* = 1.2) effects, and partial eta‐squared, with values representing small (*η*_*p*_^2^ = 0.10), medium (*η*_*p*_^2^ = 0.25), and large (*η*_*p*_^2^ = 0.40) effects, were used to assess effect sizes. To investigate the relationship between laboratory‐derived measures in PRE, the percentage change from PRE to POST, as well as decoupling characteristics, and marathon performance, Pearson product‐moment correlation coefficient was used, with 0.1–0.3 representing small, 0.3–0.5 representing medium, and 0.5–1.0 representing large strengths of association. To control for multiple tests, Holm–Bonferroni correction was applied to the *p*‐values of Pearson correlation analyses where significant associations with marathon performance were observed. Significance level was set at *p* < 0.05.

## 3 Results

### 3.1 Participants and Prolonged Trial Characteristics

Of the 23 participants who started the study, 18 runners (11 men, 7 women, Age: 41 ± 12 years, stature: 1.79 ± 0.07 m, mass: 72.6 ± 10.4 kg, running volume: 76.2 ± 24.3 km·wk^−1^) completed both laboratory visits and the 2024 London Marathon. One participant did not complete the marathon due to injury; three participants completed the first laboratory visit only and subsequently withdrew, and one participant could not complete the prolonged trial due to injury. Therefore, their data was excluded from the analysis. Participants completed the 90 min run at 12.8 ± 2.0 km·h^−1^, covering an average of 19.2 ± 3.0 km (range: 14.6–25.8 km). Body mass decreased significantly throughout the long run from 64.6 ± 12.4 to 63.2 ± 12.0 kg (delta −1.4 ± 0.7 kg, perc −2.1% ± 0.9%, *p* < 0.001). Mean total water consumption during the prolonged run was 0.50 ± 0.29 L. Significant main effects of time (all *p* < 0.05) were present with increases in B[La], RPE, HR, energy expenditure, carbohydrate oxidation, *F*_*R*_, %V̇O_2peak_, and decreases in RER, *V*_*T*_, fat oxidation were also evident (Table 1). However, no significant difference between time points was noted for *V*_*E*_ (*p* = 0.068). The energetic cost of running showed a main effect for time (*p* = 0.034), but subsequent post hoc correction demonstrated no significant changes (*p* > 0.05).

**TABLE 1:** Changes to B[La], RPE, HR, substrate utilisation, ventilatory parameters and running economy during the 90‐min run.

	15 min	30 min	60 min	90 min	Effect size (*η*_*p*_^2^)	*F* value
B[La] (mmol·L^−1^)	—	1.94 ± 0.70^a,b^	2.21 ± 0.73^a,c^	2.58 ± 0.84^b,c^	0.568	23.306
RPE (6–20)	—	11.9 ± 1.5^a,b^	12.7 ± 1.3^a,c^	13.9 ± 1.6^b,c^	0.681	36.217
HR (beats·min^−1^)	153 ± 10^a,b,c^	158 ± 10^a,d,e^	161 ± 10^b,d^	163 ± 10^c,e^	0.574	22.885
Substrate utilisation						
RER	0.94 ± 0.04^a^	0.93 ± 0.03^b^	0.91 ± 0.03^a,c^	0.90 ± 0.02^a,b,c^	0.366	9.814
FatOx (g·min^−1^)	0.30 ± 0.16^a,b^	0.32 ± 0.17^c^	0.39 ± 0.16^a,d^	0.44 ± 0.14^b,c,d^	0.383	10.556
CarbOx (g·min^−1^)	2.69 ± 0.63^a^	2.63 ± 0.70^b^	2.49 ± 0.70^c^	2.35 ± 0.68^a,b,c^	0.368	9.902
Ventilatory parameters						
*V̇*_*E*_ (L·min^−1^)	82.2 ± 18.3	83.3 ± 18.7	83.8 ± 18.6	85.5 ± 19.0	0.129	2.519
*F*_*R*_ (breaths·min^−1^)	44.5 ± 7.2^a,b^	47.5 ± 8.8^a,c^	47.9 ± 8.2^d^	51.0 ± 10.4^b,c,d^	0.403	11.493
*V*_*T*_ (L·breath^−1^)	1.87 ± 0.41^a^	1.79 ± 0.48^b^	1.79 ± 0.48^c^	1.72 ± 0.46^a,b,c^	0.380	10.413
Running economy						
RE (ml·kg^−1^·km^−1^)	202.4 ± 16.4	203.0 ± 14.1	205.8 ± 15.4	206.2 ± 14.1	0.130	2.541
EE (kcal·kg^−1^·km^−1^)	1.01 ± 0.07	1.02 ± 0.08	1.02 ± 0.08	1.03 ± 0.07	0.155	3.108

*Note:* Matching superscript letters denote significant differences between corresponding time points in the same row (*p* > 0.05).

Abbreviations: B[La], blood lactate concentration; CarbOx, carbohydrate oxidation rate; EE, whole body energy expenditure. FatOx, fat oxidation rate; *F*_*R*_, breathing frequency; HR, heart rate; RE, running economy; RER, respiratory exchange ratio; RPE, rating of perceived exertion (Borg 1998); *V̇*_*E*_, rate of ventilation; *V*_*T*_, tidal volume.

### 3.2 Changes in Determinants of Marathon Performances

**FIGURE 2:**
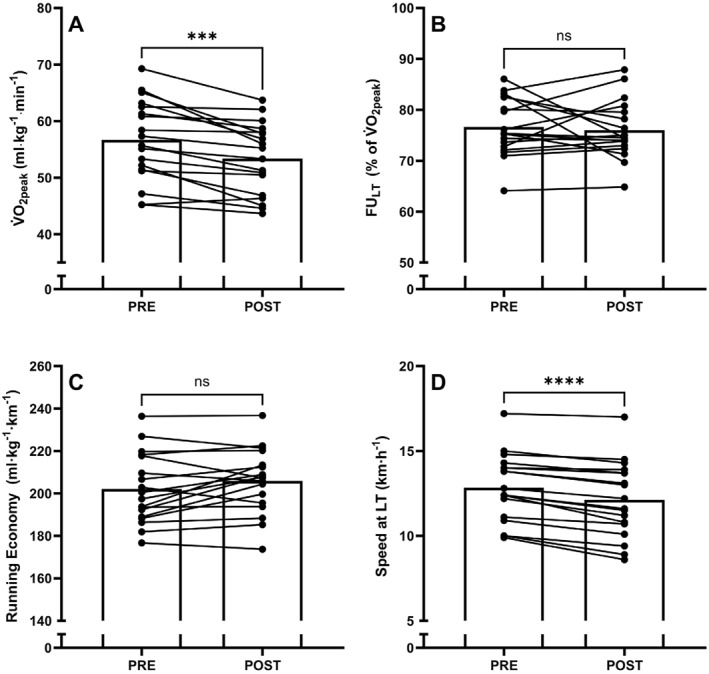
Changes in (A) peak oxygen uptake (V̇O_2peak_), (B) the fractional utilisation of V̇O_2peak_ at lactate threshold (FU_LT_), (C) running economy and (D) the speed at lactate threshold (LT), between PRE and POST assessments. Asterisks denote a significant difference between assessments where *** denotes *p* < 0.001, **** denotes *p* < 0.0001, and ns denotes no significant difference (*N* = 18).

Following prolonged running, sLT, relative and absolute V̇O_2_ at LT, energy expenditure, and substrate utilisation at LT all changed significantly when compared to the PRE assessment (*p* < 0.001, Table 1, Figure 2). Specifically, sLT decreased from 12.8 ± 2.0 km·h^−1^ in PRE to 12.1 ± 2.2 km·h^−1^ in POST (*p* < 0.001, *d* = 1.840), and relative V̇O_2_ at LT decreased from 43.4 ± 6.0 mL·kg^−1^ min^−1^ in PRE compared to 40.7 ± 6.5 mL·kg^−1^ min^−1^ in POST (*p* < 0.001, *d* = 1.157). No significant changes between PRE and POST were observed in FU_LT_ (PRE: 76.6% ± 5.7% vs. POST: 76.0 ± 5.7%, *p* = 0.644, *d* = 0.111). Decreases were observed in both relative V̇O_2peak_ (PRE: 56.7 ± 7.2 mL·kg^−1^ min^−1^ vs. POST: 53.4 ± 6.3 mL·kg^−1^ min^−1^, *p* < 0.001, *d* = 1.127) and absolute V̇O_2peak_ (PRE: 3.64 ± 0.81 L·min^−1^ vs. POST: 3.37 ± 0.71 L·min^−1^, *p* < 0.001, *d* = 1.395) when measured in the POST condition compared to PRE condition. RE showed no significant change between 15 and 90 min (*p* = 0.081).

### 3.3 Marathon Performance and Association With IET Parameters

**FIGURE 3:**
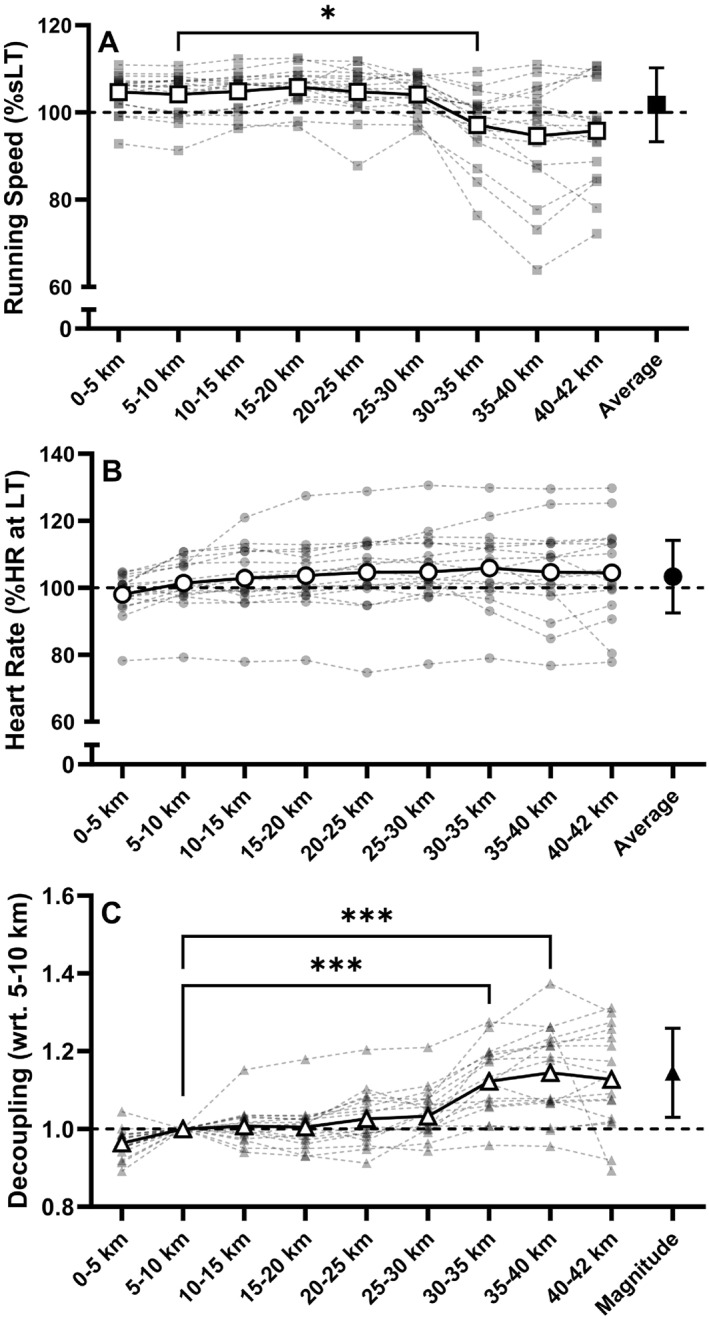
Changes to (A) speed, (B) heart rate and (C) decoupling during the marathon (*N* = 17). sLT, speed at lactate threshold (LT) measured in fresh conditions; HR at LT, heart rate at LT in fresh conditions. Mean values for each parameter are given in white shapes with connecting lines. Individual participant data are given in grey with dotted lines between consecutive segments. The overall group mean running speed and heart rate attained throughout the marathon, and mean decoupling magnitude at 35–40 km are given in black, with error bars representing standard deviation. Pairwise comparisons represent significant differences from the 5–10 km segment, where * denotes *p* < 0.05 and *** denotes *p* < 0.001 (*N* = 17).

Participants completed the marathon in 3:17 ± 0:32 h:min (range: 2:27–4:14), at 13.1 ± 2.1 km·h^−1^ (range: 10.0–17.2 km·h^−1^). HR data from the marathon was missing from one participant due to technical issues with the sports watch. Figure 3 illustrates individual and group speed relative to sLT, HR relative to the HR at LT, and the decoupling of the two parameters in 17 participants. The average magnitude of HR‐to‐speed decoupling was 1.14 ± 0.11 a.u., and decoupling was first observed at 26.9 ± 10.1 km. Neither the magnitude (*r* = −0.058, *p* = 0.826) or onset (*r* = −0.067, *p* = 0.798) of HR‐to‐speed decoupling were associated with marathon performance. The magnitude of decoupling during the marathon was not associated with differences from PRE to POST assessments (Table S1).

**FIGURE 4:**
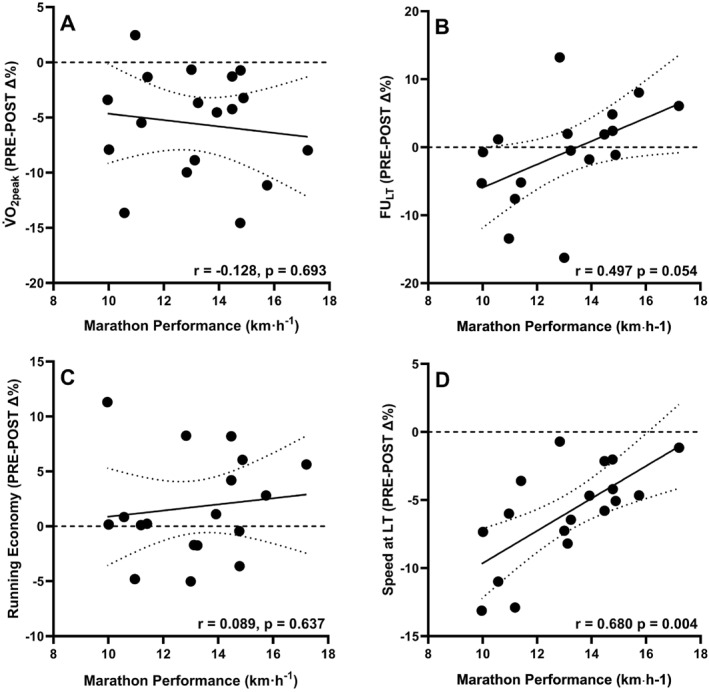
Relationships between marathon performance and the percentage change in parameters from PRE to POST assessments (PRE‐POST Δ%) in (A) peak oxygen uptake (V̇O_2peak_), (B) the fractional utilisation of V̇O_2peak_ at lactate threshold (FU_LT_), (C) running economy and (D) the speed at lactate threshold (LT) (*N* = 18). *p* values adjusted for multiple comparisons using the Holm‐Bonferroni method.

Marathon performance was significantly associated with V̇O_2peak_ and RE, but not FU_LT_ (Table 2). Although the percentage change in FU_LT_ from PRE to POST was initially significantly correlated with marathon performance (*p* = 0.018), this association did not remain statistically significant (*p* = 0.054) after applying Holm‐Bonferroni correction for multiple comparisons (Table 2, Figure 4). The sLT in PRE (*r* = 0.973, *p* < 0.001) and the percentage change in sLT from PRE to POST (*r* = 0.680, *p* < 0.001) were also significantly correlated with marathon performance (Figure 4). Significant associations for sLT in PRE (*p* < 0.001) and the percentage change in sLT from PRE to POST (*p* = 0.003) both remained following Holm‐Bonferroni correction for multiple comparisons.

**TABLE 2:** Associations between key physiological measured in the PRE assessment, the percentage difference between PRE and POST measures, and marathon performance (*N* = 18).

	Marathon performance from fresh measures	Marathon performance from % diff. between PRE and POST
Relative V̇O_2peak_	0.809*** [0.549, 0.926]	−0.128 [−0.561, 0.361]
FU_LT_	−0.102 [−0.543, 0.383]	0.497 [0.039, 0.782]
RE	−0.471* [−0.769, −0.006]	0.125 [−0.358, 0.563]
sLT	0.937*** [0.835, 0.977]	0.680 ** [0.312, 0.871]

*Note:* Values represent correlation coefficients, with values in square brackets denoting 95% confidence intervals. Asterisks denote a significant association between parameters and marathon performance where * *p* < 0.05, **p < 0.01, and ***p < 0.001. Relative V̇O_2peak_, peak oxygen uptake relative to body mass; FU_LT_, fractional utilisation of V̇O_2peak_ at LT; RE, running economy; sLT, speed at lactate threshold.

## 4 Discussion

This is the first evidence to demonstrate that a laboratory‐based assessment of durability is associated with marathon performance. Specifically, the durability of the lactate threshold, quantified as the percentage change in sLT between the PRE and POST assessments, was significantly correlated with marathon performance (Table 2, Figure 4). Relative to PRE, decreases in sLT and V̇O_2peak_ were observed following a 90‐min run. Throughout the 90‐min run, increases in B[La], RPE, HR, energy expenditure, carbohydrate oxidation, *F*_*R*_, %V̇O_2peak_, and decreases in RER, *V*_*T*_, fat oxidation were evident (Table 3). However, changes to energy expenditure were not observed, no changes to RE were evident during prolonged running, and the decoupling of the HR‐to‐speed ratio was not associated with performance.

**TABLE 3:** Comparison of variables at lactate threshold (LT) in PRE and POST (*N* = 18).

	PRE	POST	Effect size (*d*)
sLT (km·h^−1^)***	12.8 ± 2.0	12.1 ± 2.2	1.840
V̇O_2_ at LT (L·min^−1^)***	2.78 ± 0.61	2.57 ± 0.63	1.617
V̇O_2_ at LT (ml·kg^−1^ min^−1^)***	43.4 ± 6.0	40.7 ± 6.5	1.157
FU_Lt_ (%)	76.6 ± 5.7	76.0 ± 5.7	0.111
HR (beats·min^−1^)	156 ± 10	154 ± 11	0.253
EE (kcal·kg^−1^·km^−1^)**	1.08 ± 0.19	1.06 ± 0.20	0.708
FatOx (g·min^−1^)***	0.26 ± 0.16	0.52 ± 0.15	1.316
CarbOx (g·min^−1^)***	2.87 ± 0.63	1.90 ± 0.62	2.450
*V̇*_*E*_ (L·min^−1^)**	85.7 ± 18.0	78.4 ± 18.4	0.844
*F*_*R*_ (breaths·min^−1^)***	44.5 ± 7.3	48.6 ± 8.5	0.906
*V*_*T*_ (L·breath^−1^)***	1.95 ± 0.36	1.65 ± 0.37	2.025

*Note:* Asterisks denote a significant difference between assessments where *p < 0.05, **p < 0.01, and ***p < 0.001.

Abbreviations: CarbOx, carbohydrate oxidation rate; EE, whole body energy expenditure; FatOx, fat oxidation rate; *F*_*R*_, breathing frequency; HR, heart rate; sLT, speed at lactate threshold; *V̇*_*E*_, rate of ventilation; *V*_*T*_, tidal volume.

### 4.1 Lactate Threshold and Fractional Utilisation of V̇O_2max_

Concurrent with previous observations (Barrett and Maunder 2025; Nuuttila et al. 2024; Zanini et al. 2025), it was found that the speed at the moderate‐to‐heavy boundary was reduced following prolonged running. However, there was no change in FU_LT_ between PRE and POST. This contrasts with the work of Zanini et al. (2025), where an increase in FU_LT_ from 78.6% to 80.4% of V̇O_2peak_ was observed after a 90‐min heavy‐domain run, mainly due to a reduced V̇O_2peak_ with unchanged V̇O_2_ at LT. In the current study, both reductions in V̇O_2peak_ and reductions in V̇O_2_ at LT were evident, but did not occur at the same rate within participants. As such, eight participants exhibited increased FU_LT_, but this did not represent an improved fraction of aerobic capacity which is accessible during endurance exercise. Indeed, the current study demonstrated a mean reduction in the V̇O_2_ at LT by 2.7 ± 2.3 mL·kg^−1^ min^−1^, with only three participants demonstrating an increase in V̇O_2_ at LT, by an average of 0.63 mL·kg^−1^ min^−1^. Therefore, following prolonged exercise, reporting the V̇O_2_ associated with physiological thresholds in absolute terms (ml·kg^−1^ min^−1^), rather than relative to maximal values (e.g., % of V̇O_2peak_), may provide a more accurate indication of the durability of the ‘performance’ V̇O_2_. Indeed, when compared to the durability of FU_LT_ (*r* = 0.497, *p* = 0.108), the durability of the relative V̇O_2_ at LT showed a slightly stronger association with marathon performance which remained significant after correcting for multiple comparisons (*r* = 0.534, *p* = 0.034).

Towards the start of the prolonged run, runners exercised at 77.2% ± 5.6% of V̇O_2peak_, increasing throughout the prolonged run, ending at 82.4% ± 5.3% of V̇O_2peak_, indicating an upward shift in intensity, as reflected in the observed increase in B[La] and RPE. This finding has important implications for load management and exercise prescription: a relatively short 90‐min run is enough to create an upward shift in exercise intensity. Future research should explore the specific time points at which LT shifts in running, as well as the effects of different run durations and training statuses. Previously, the onset of these changes has been shown to vary between individuals (Gallo et al. 2024). A further understanding of these dynamics in running could help optimise training protocols, monitoring, and performance profiling.

The experimental approach does not allow for a thorough investigation into the mechanisms behind the decrease in V̇O_2_ at LT, and this warrants further investigation. However, the decrease in V̇O_2_ at LT coincided with a decreased rate of energy expenditure and carbohydrate oxidation (Table 3), indicating reduced glycogen availability. This may have contributed to diminished contractile function of predominantly oxidative fibres, as shown previously by Nielsen et al. (2024), and a greater reliance on less‐oxidative fibres. The greater reliance on less‐oxidative fibres may also explain the increase in B[La] observed throughout the prolonged run. Further work is required to understand the role of glycogen depletion, and other mechanistic underpinnings, on the downward shift in LT following prolonged exercise.

### 4.2 V̇O_2peak_

Runners exhibited a ∼6% reduction in V̇O_2peak_ in the POST condition when compared to PRE. Data from previous studies utilising a similar duration (Dressendorfer 1991; Zanini et al. 2025) have demonstrated similar magnitudes of change, although no change in V̇O_2max_ has been observed following a 60‐min run (Unhjem 2024). Despite a less intense and shorter protocol adopted in the current study, the rate of decline is similar to that shown by Zanini et al. (2025) following a 120‐min run in the heavy domain, where a ∼7% reduction was evident. This similarity, despite the difference in time and intensity, may be due to the lack of carbohydrate supplementation used in the current study. Carbohydrate availability has been shown to attenuate the decline in physiological function during prolonged exercise (Clark et al. 2019). In the absence of exogenous carbohydrate intake, reduced glycogen availability may have occurred earlier, contributing to a premature reduction in V̇O_2peak_. Indeed, carbohydrate oxidation reduced throughout the course of the prolonged run, indicating glycogen depletion. Further to the effects of glycogen depletion, participants decreased body mass by 2.1% throughout the run, alongside elevated HR at the end of the 90‐min run, suggesting dehydration. Declines in stroke volume, and a subsequent reduction in cardiac output, caused by dehydration may have also contributed to a reduction in V̇O_2peak_, as shown previously (Ganio et al. 2006). Another plausible hypothesis is that the ability to attain a similar V̇O_2peak_ to baseline could be predicated on a psychological aspect. For example, the ability to perform work at high intensities has been shown to be impacted by pre‐task and in‐task motivation (Taylor et al. 2020). In the context of a maximal IET, a lack of motivation to complete high intensity exercise coupled with increased perception of effort at the end of the prolonged run may result in a diminished ability to attain a similar V̇O_2peak_ to baseline.

### 4.3 Running Economy

Contrary to the hypothesis and findings of previous literature (Brueckner et al. 1991; Kyröläinen et al. 2000; Nicol et al. 1991; Nuuttila et al. 2024; Unhjem 2024; Zanini et al. 2024), RE showed no significant change during prolonged exercise. This was unexpected given the increased reliance on fat oxidation demonstrated throughout the prolonged run, which is associated with a greater V̇O_2_ for a given rate of energy production (Jeukendrup and Wallis 2005). Although this cannot be determined from the current dataset, the absence of change in running economy RE may reflect a reduced reliance on less efficient type II muscle fibres over time (Vollestad and Blom 1985), along with minimal biomechanical alterations as previously suggested (Zanini et al. 2025). However, the influence of shifts in muscle recruitment patterns and biomechanics on RE during prolonged exercise warrants further investigation. A 2% change in relative EE was observed throughout the prolonged run, lower than the 3.4% change observed by Zanini et al. (2024) in 51 male runners, but no significant difference was detected. The discrepancies in changes to EE may be due to the comparatively smaller sample size used in the current study, as well as the recruitment of both men and women, which have previously shown to exhibit differences in energy expenditure during prolonged exercise (Nuuttila et al. 2024).

It has previously been suggested that changes as a function of distance, rather than time, allow for more robust comparisons between athletes, as it is not confounded by differences in running speed (Zanini et al. 2024). Despite differences in running speed between the studies, no significant difference in RE has been found after 15 km (Brueckner et al. 1991), 22.3 km (Barrett and Maunder 2025), 21 km (Nicol et al. 1991) and 16.7 km in a group of high performing runners (Zanini et al. 2024). It is noteworthy the runners studied by Brueckner et al. (1991) and Nicol et al. (1991) exhibited a deterioration of RE, but only after 32 and 42 km, respectively. Although the underpinnings of durability remain unresolved, it has been suggested that higher training volumes may promote durability of RE (Zanini et al. 2024). We posit that the specificity of training undertaken by the cohort in the current study resulted in sufficient durability of RE. When coupled with the relatively short running bout, covering 19.2 ± 3.0 km, this was not sufficient to elicit significant changes in RE in the current group. Given the conflicting findings between studies, further work is warranted to explore changes to RE and EE during prolonged exercise, especially as a result of marathon‐specific training.

### 4.4 Decoupling

During the marathon, significant changes to decoupling were evident following ∼70% of the total distance of the marathon, similar to onset of decoupling (∼60%) shown by Smyth et al. (2022). Consistent with previous observations (De Pauw et al. 2024; Smyth et al. 2022), this decoupling was caused primarily through a decrease in speed, rather than an upward drift in HR. Contrary to existing evidence, however, HR‐to‐speed decoupling was not associated with running performance, or with laboratory‐based measures of durability. The runners studied by De Pauw et al. (2024) completed a backyard ultramarathon, and may have been better able to rehydrate, limiting cardiac drift due to hypovolaemia. The size of the dataset (> 80,000 runners) used by Smyth et al. (2022), which found an association between decoupling and marathon performance, likely masked ‘noise’ of anomalous results. However, the small sample size adopted in the current study may have been impacted by a multitude of extraneous factors which could not have been controlled for including dehydration, maladaptive pacing strategies, differences in equipment used to measure HR, and cognitive changes such as arousal. Further work investigating more controlled conditions (e.g., Rothschild et al. (2025)) should be carried out to examine the efficacy of decoupling as a potential tool to monitor durability in running.

### 4.5 Associations With Marathon Performance

Consistent with previous observations (di Prampero et al. 1986), two of the traditional three pillars of endurance performance, RE and V̇O_2peak_, demonstrated significant associations with marathon performance (Table 2). However, despite a wide range (64%–86%), FU_LT_ was not related to marathon speed. The lack of association between FU_LT_ is likely explained by the fact that this parameter should be considered alongside V̇O_2peak_, where the product of the two dictate the ‘performance’ V̇O_2_ (Jones et al. 2021; Joyner 1991). Indeed, the notion of FU_LT_ as a determinant of endurance performance has been challenged, with no differences being demonstrated between elite and regional endurance athletes (Johansen et al. 2025). The percentage decrease in V̇O_2peak_ following the 90‐min run was not significantly associated with marathon performance. Although V̇O_2peak_ represents the upper limit for aerobic metabolism, it should be considered alongside the fractional utilisation of V̇O_2peak_ at thresholds for marathon performance (di Prampero et al. 1986; Jones et al. 2021; Joyner and Coyle 2008). This may explain the lack of association between changes to V̇O_2peak_ from PRE to POST and marathon performance.

No significant associations between the durability of FU_LT_ marathon performance were demonstrated. However, the durability of the relative V̇O_2_ at LT, potentially a better indication of the ‘performance’ V̇O_2_, was significantly related with marathon performance. A large strength of association was also shown between marathon performance and the durability of sLT. The downward shift of physiological thresholds represents a transition into a higher intensity domain, resulting in greater physiological stress (Black et al. 2017). Although speculative, a greater downward shift in sLT observed following the 90‐min run may therefore correspond to: (i) an increased rate of glycolysis (Cannon et al. 2014) that contributed to premature depletion of glycogen, and (ii) more severe impairments in neuromuscular function (Brownstein et al. 2022) during the marathon, resulting in diminished performance. As the sLT is comprised of the V̇O_2peak_, FU_LT_, and RE, assessing its durability offers a composite measure of changes across these key physiological parameters. Notably, the extent of decline in each component differed between runners, highlighting the potential value of sLT durability as an integrative performance metric.

The association between durability and marathon performance represents an important finding, which confirms the notion that durability should be considered as an ‘additional component’ or the ‘fourth dimension’ of endurance exercise performance (Jones 2023). It is worth noting that the associations between durability and marathon performance were weaker than that of some traditional markers (e.g., sLT (*r* = 0.937) and the percentage change in sLT (*r* = 0.680). If we consider the current dataset, sLT values ranged from 9.9 km·h^−1^–17.2 km·h^−1^. When comparing two runners with sLT of 9.9 km·h^−1^ and 17.2 km·h^−1^, whether their sLT decreases by 0.7% or 13.1% would have little impact when compared to the substantial differences in their initial physiological capacity. Therefore, as previously suggested (Jones 2023), durability may be of greater relevance when differentiating performance capabilities of athletes with similar baseline physiological characteristics. Although challenging, a study design examining performances of a group exhibiting similar baseline physiological characteristics, and the subsequent downward shift in these characteristics may shed further light on the concept of durability.

### 4.6 Limitations

The intensity used in the study was set at the speed of LT to provide a similar stimulus to marathon speed whilst anchoring it to a common physiological landmark. Indeed, the speed of the fatiguing protocol was ∼99% (98.7% ± 6.4%) of the participants' average speed during the marathon. However, when accounting for measurement error, this may have resulted in some participants exercising in the heavy domain or moderate domain. Although the log‐log method was used to determine LT, which is less dependent on absolute lactate values, the absence of a blood lactate measurement immediately prior to the POST assessment may have influenced the estimation. Residual elevation in lactate following the 90‐min run could have shifted the lactate‐speed curve, potentially affecting the measurement of LT in the POST condition. The distance covered during the prolonged running bout performed in the laboratory (45.6% ± 7.1% of marathon distance) was shy of the halfway mark for the marathon. It could be that if the prolonged running were longer, this could further improve the strength of associations between durability and marathon performance. However, due to the proximity of the testing to the marathon, it may have reduced the number of participants willing to take part in the study. Finally, endurance performance is affected by dietary intake. Despite informing participants to keep dietary intake consistent between visits, it was not controlled.

## 5 Conclusions

Consistent with previous investigations, reductions in V̇O_2peak_ and sLT were observed following a prolonged run, but no significant differences were shown in RE or FU_LT_. The change in sLT from PRE to POST was significantly associated with marathon performance, with smaller deteriorations associated with faster marathon times. Prolonged running impairs key physiological markers of endurance performance, and the degree of this deterioration, that is, durability, is associated with marathon performance. Therefore, it may be important for marathon runners and practitioners to consider durability when conducting physiological profiling.

## Conflicts of Interest

The authors declare no conflicts of interest.

## Supporting information


**Table S1**: Associations between the HR‐to‐speed decoupling experienced during the marathon and the percentage difference between PRE and POST measures (*N* = 17). CarbOx, carbohydrate oxidation rate; EE, whole body energy expenditure; FatOx, fat oxidation rate; *F*_*R*_, breathing frequency; FU_LT_, fractional utilisation of V̇O2peak at lactate threshold; HR, heart rate; LT, lactate threshold; RE, unning economy; sLT, speed at lactate; *V̇*_*E*_, rate of ventilation; V̇O2peak, peak oxygen uptake; *V*_*T*_, tidal volume.
