# Supplementary material for: Durability of Parameters Associated With Endurance Running in Marathoners
Source: Eur J Sport Sci. 2025 Oct 23;25(11):e70073. doi: 10.1002/ejsc.70073 (PMC12547624; doi:10.1002/ejsc.70073)
Supplement: Supplementary file 1 — Table S1: Associations between the HR‐to‐speed decoupling experienced during the marathon and the percentage difference between PRE and POST measures (N = 17). CarbOx, carbohydrate oxidation rate; EE, whole body energy expenditure; FatOx, fat oxidation rate; F R , breathing frequency; FULT, fractional utilisation of V̇O2peak at lactate threshold; HR, heart rate; LT, lactate threshold; RE, unning economy; sLT, speed at lactate; V̇ E , rate of ventilation; V̇O2peak, peak oxygen uptake; V T , tidal volume. [file EJSC-25-e70073-s001.docx]

Table S1. Associations between the HR-to-speed decoupling experienced during the marathon and the percentage difference between PRE and POST measures (N = 17).

| FU_LT_ | 0.438 |
| --- | --- |
|  | [-0.054, 0.759] |
|  | p = 0.079 |
| sLT | -0.133 |
|  | [-0.577, 0.371] |
|  | p = 0.610 |
| V̇O_2peak_ | -0.393 |
|  | [-0.735, 0.108] |
|  | p = 0.118 |
| EE at LT | 0.205 |
|  | [-0.306, 0.624] |
|  | p = 0.43 |
| HR at LT | 0.343 |
|  | [-0.165, 0.707] |
|  | p = 0.178 |
| V̇_E_ at LT | 0.344 |
|  | [-0.163, 0.708] |
|  | p = 0.176 |
| V_T_ at LT | 0.15 |
|  | [-0.356, 0.588] |
|  | p = 0.565 |
| F_R_ at LT | 0.168 |
|  | [-0.340, 0.600] |
|  | p = 0.520 |
| FatOx at LT | -0.112 |
|  | [-0.562, 0.390] |
|  | p = 0.669 |
| CarbOx at LT | 0.308 |
|  | [-0.203, 0.687] |
|  | p = 0.229 |
| V̇O_2_ at LT | 0.181 |
|  | [-0.328, 0.609] |
|  | p = 0.486 |
| RE | 0.161 |
|  | [-0.347, 0.595] |
|  | p = 0.538 |
| Estimated Marathon Speed | 0.079 |
|  | [-0.418, 0.539] |
|  | p = 0.763 |
| Magnitude of HR-to-Speed Decoupling in Prolonged Laboratory Trial | 0.098 |
|  | [-0.402, 0.552] |
|  | p = 0.709 |

FU_LT_, fractional utilisation of V̇O_2peak_ at lactate threshold; sLT, speed at lactate; LT, lactate threshold; V̇O_2peak_, peak oxygen uptake; HR, heart rate; EE, whole body energy expenditure; FatOx, fat oxidation rate; CarbOx, carbohydrate oxidation rate; V̇_E_, rate of ventilation; F_R_, breathing frequency; V_T_, tidal volume; RE, running economy.
